# Supplementary material for: Content-rich biological network constructed by mining PubMed abstracts
Source: BMC Bioinformatics. 2004 Oct 8;5:147. doi: 10.1186/1471-2105-5-147 (PMC528731; doi:10.1186/1471-2105-5-147)
Supplement: Additional File 2 — The original results of the above study (non-essential files are deleted to keep the file size under the limit set by BMC bioinformatics). [file 1471-2105-5-147-S2.bz2 › chilibotAdditionalFile2/dip30/668ID9122217E11068/html/TRAF3_LTBR.html]

 


 **TRAF3** and **LTBR** 
  
Found 22 abstracts in PubMed, retrieved 22.  
 

 What does Google say? 
 PDF only 
| .edu only 

---

**Interactive relationship** (e.g. stimulation, inhibition, etc)

**Neutral relationship**- Ligation of the lymphotoxin beta receptor  [ **LTBR** ]  LTbetaR recruits tumor necrosis factor receptor associated factor 3  **TRAF3**  and initiates cell death in HT29 adenocarcinoma cells.  Ref: 9388227 J Biol Chem, 1997
- The Epstein Barr virus BNLF 1 membrane protein LMP1  [ **TRAF3** ]  induces homotypic adhesion mediated by CD11a CD18  [ **LTBR** ]  in a murine B cell line, mimicking the action of phorbol ester.  Ref: 1680199 J Virol, 1991
- CD2 was shown to syn cap  [ **TRAF3** ]  unidirectionally cocap with CD44 and CD11a CD18  [ **LTBR** ]  LFA 1, an observation potentially related to functional cooperation among these molecules in T cell activation.  Ref: 7678845 J Leukoc Biol, 1993
- LAP1  [ **TRAF3** ]  binds to the p80 TNFR, CD40, and the lymphotoxin beta receptor  [ **LTBR** ] , while EBI6 associates with the p80 TNFR.  Ref: 7859281 Cell, 1995
- Dominant negative mutants of  **TRAF3**  reveal an important role for the coiled coil domains in cell death signaling by the lymphotoxin beta receptor  [ **LTBR** ] .  Ref: 9388227 J Biol Chem, 1997
- TRAFamn  [ **TRAF3** ]  is identical to a recently reported protein CD40bp  [ **TRAF3** ] , CAP 1  [ **TRAF3** ] , CRAF1  [ **TRAF3** ] , LAP1  [ **TRAF3** ]  that can bind the cytoplasmic domains of CD40 and the lymphotoxin beta receptor  [ **LTBR** ]  LTbetaR, both of which are known members of the TNF receptor superfamily.  Ref: 8660894 Dev Biol, 1996

**Non-interactive relationship** (e.g. studied together, co-existance, homology, etc.)

- We investigated 49 acquired immunodeficiency syndrome related lymphomas ARLs for Epstein Barr virus EBV by Southern blotting and in situ hybridization and, in positive cases, used cryostat immunohistology to compare EBV latent gene expression EBV encoded small RNA 1 EBER 1, EBV nuclear antigen 2 EBNA 2, latent membrane protein 1 LMP 1  [ **TRAF3** ]  and host cell immunophenotype CD11a, CD18  [ **LTBR** ] , CD54, CD58, CD21, CD23, CD30, CD39, CDw70, immunoglobulin patterns with those reported in other EBV infections.  Ref: 8214003 Am J Pathol, 1993
- Endogenous association of TRAF2,  **TRAF3** , cIAP1, and Smac with lymphotoxin beta receptor  [ **LTBR** ]  reveals a novel mechanism of apoptosis.  Ref: 12571250 J Biol Chem, 2003
- TRAF6 does not associate with the cytoplasmic tails of TNFR2, CD30, lymphotoxin beta receptor  [ **LTBR** ] , and LMP1  [ **TRAF3** ]  of Epstein Barr virus.  Ref: 8910514 J Biol Chem, 1996
- It takes part in the signal transduction of the TNF receptor 2 TNFR2, the lymphotoxin beta receptor  [ **LTBR** ]  LT betaR, CD40, CD30, and LMP1  [ **TRAF3** ] .  Ref: 9885224 Blood, 1999
- Cytochalasin D 10 5 M produced CD18  [ **LTBR** ]  accumulation at the pole opposite the F actin cap  [ **TRAF3** ] .  Ref: 7904268 J Cell Sci, 1993
